# Screening for the risk of canine impaction, what are the presumptive signs and how does it affect orthodontics? A cross-sectional study in France

**DOI:** 10.1371/journal.pone.0296395

**Published:** 2023-12-29

**Authors:** Damien Brézulier, Steeven Carnet, Alexia Marie-Cousin, Jean-Louis Sixou

**Affiliations:** 1 CHU Rennes, Pôle Odontologie, Univ Rennes, Rennes, France; 2 ISCR UMR 6226, Univ Rennes, Rennes, France; University of Catanzaro, ITALY

## Abstract

**Purpose:**

The treatment of impacted canines is a challenge for orthodontists. The availability of suggestive clinical signs has become crucial for treatment before the potential for evolution ceases. The main objective was to evaluate the prevalence of the suspected displaced canine (SDC) and to highlight factors easily identifiable from the oral examination.

**Methods:**

SDC was assessed on panoramic X-rays, on the basis of the angle with the median sagittal plane and the degree of overlap with the permanent lateral incisor. Its association with mesio-distal tooth and palate widths was assessed by univariate analysis. Next, the association of SDC with temporary tooth extraction, expansion and/or premolar extraction was considered using the same modality.

**Results:**

In this retrospective study, the records of 292 patients aged 7 to 13 years were reviewed. SDC was detected in 39% of patients i.e., 28,8% of observed canines. Reduced coronal mesio-distal diameters of permanent maxillary central incisors, 8.7 ± 0.6 mm versus 8.8 ± 0.7 mm (p < .05), and first permanent molars, 10.0 ± 0.7 mm versus 10.2 ± 0.7 mm (p < .001), as well as reduced inter-molar width, 38.7 ± 2.7 mm versus 39.4 ± 2.9 mm (p < .01), were predictive factors. SDC led orthodontists to indicate extraction of maxillary primary canines, OR = 3,32 (p < .001) or even extraction of premolars, OR = 1,66 (p < .05).

**Conclusion:**

This study confirmed the interest of panoramic X-rays in detected canines at risk of SDC. Dental factors can be combined to make screening more reliable and predict impaction that makes orthodontics complex.

**Trial registration number:**

Opinion n°21.131, dated 09.21.2021, retrospectively registered.

## Introduction

Maxillary permanent canines normally erupt between the ages of 10 and 12 years. Tooth impaction can be defined as the infraosseous position of the tooth after the expected time of eruption, whereas the anomalous infraosseous position of the canine before the expected time of eruption can be defined as a displacement [1]. The consequences of incorrect canine placement range from lack of functional occlusion to temporomandibular joint disorders in adulthood [2].

They are the teeth at higher risk of impaction after wisdom teeth: 1 to 3% in the general population [3–5]. The etiology of maxillary canine impaction remains poorly understood. Affected siblings suggest a hereditary component (genetic theory) [6]. Canine impaction can also occur due to local environmental factors related to agenesis, anatomic abnormalities or late development of adjacent lateral incisors that cannot guide canine eruption (theory of palatal canine movement guidance) [7, 8].

The lack of clinical symptoms may delay diagnosis and therefore complicate management [9]. Generally speaking, impaction is detected by chance during a routine dental examination. However, several warning signs may suggest or indicate a problem with the eruption of these teeth. In this case we speak of suspected displaced canine (SDC). Signs can be either clinical—maxillary anterior crowding or transverse discrepancy—or radiographic. Radiographically, deviation of the canine eruption axis or obstacles to eruption (odontoma, supernumerary tooth) suggest SDC [10]. The evaluation of the risk of impaction depends on various parameters on the orthopantomogram: inclination of the maxillary canine to the midline, distance to the occlusal plane or position of the crown in relation to the lateral incisor [11].

An early diagnosis of SDC is therefore crucial to allow better prevention and interception [12]. Management of impacted teeth requires a multidisciplinary cooperation between orthodontists, oral surgeons and sometimes periodontists, which increases the duration of orthodontics. Beyond the economic consequences, the risks associated with this therapy are numerous, including lesions of the adjacent teeth [13]. The objective of this study was, initially (primary outcome), to evaluate the prevalence of SDC in a population of adolescents undergoing orthodontic treatment at the University Dental Hospital of XXX. The secondary outcomes were to identify oral factors predictive of SDC and then to evaluate orthodontic treatment choices associated with it in this cohort.

## Material and methods

### Study design and participants

A cross-sectional observational study was conducted according to the STROBE (Strengthening the Reporting of Observational Studies in Epidemiology) recommendations. The study was approved by the Ethics Committee of the University Hospital of Rennes on September 21th, 2021 (opinion n°21.131). In accordance with French regulations, the parents or legal representatives of the minors were informed orally and in writing that their healthcare data could be re-used for retrospective studies. They signed this information at the initial consultation. When the data were reused for the study, the parents of the minors were informed of the process by letter. They were sent an information letter including data management and anonymization. At the end of this campaign, the files of children whose parents had received detailed information by information letter and had responded unfavorably to inclusion were not retained. This information was added to the medical file. Data was collected between November 2, 2021 and March 10, 2022. To ensure anonymity, a first file listed the patients included. It indicated the correspondence between patient identity and file number. It should be noted that only one of the authors (SC) had access to this file. This file was entrusted to the Research and Innovation Department. A second file was used to collect data for each anonymous file number. All relevant data are within the manuscript and its S1 Dataset.

Patients who consecutively consulted for a global orthodontic assessment at University Hospital of Rennes between May 2018 and January 2021 were included in the study. The data collected came from the medical and clinical examination, panoramic X-ray and digital impressions. The inclusion criteria were: age between 7 and 13 years at the time of assessment; complete orthodontic record including models, panoramic radiographs, and treatment plan; presence of maxillary permanent central incisors and first permanent molars in the arch. The exclusion criteria were: presence of both maxillary canines on the arch; previous orthodontic treatment; syndrome, facial cleft, or rare disease influencing maxillary growth and dental development; incomplete medical record; absence of erupted permanent maxillary incisors and first molars.

### Collected data

The variables were divided into four blocks. The first block described the sample: age at assessment, gender.

The second reported the position of the two maxillary permanent canines on the orthopantomogram (sector, angulation and distance to the occlusal plane according to Ericson and Kurol’s previous works) in order to assess the risk of SDC from the angle α between the axis of the canine and the medial sagittal plane and the sector of superposition of the canine on the lateral incisor [11]. SDC was established in case of angulation strictly greater than 31° [14] or a location other than in sector 1 [15].

The third block described the maxillary dental status (presence of primary canines, resorption of permanent lateral incisors or premolars, agenesis of lateral incisors), Angle relationship, inter-molar width, and size of dental crowns (permanent central and lateral incisors, molars).

The fourth block included selected orthodontic treatment options: primary canine extractions, maxillary expansion, premolar extractions.

These data were derived from the medical record available on the Logos_w^™^ software. Measurements on x-rays were performed using the Logos_w^™^ imaging tool. Measurements on digital models were performed using the Orthoanalyzer^™^ software. All of these variables were collected in a Microsoft Excel^™^ spreadsheet by a single trained and calibrated operator (SC) who worked blindly between model and x-rays measurements.

### Statistical analysis

Statistical analysis was performed with RStudio^™^ software version 1.4.1103 (RStudioTeam) with R version 4.0.2 (RCore Team). Qualitative data were analyzed using the Pearson *χ*^2^ test. The Shapiro-Wilk test and reading of "Q-Q plots" were used to determine the normality of the distributions of quantitative data. For each group, means were compared by Student t test, after testing for the equality of variances by an F test. Univariate logistic regression analyses were performed. A p-value of ≤ 0.05 was considered statistically significant. Cohen’s kappa coefficient determined intra-rater reproducibility for quantitative and qualitative values. It was calculated on 5 cases, with readings taken at 1-month intervals.

## Results

### Intra-rater reliability

The Cohen’s kappa measurement yielded an 0.817 for quantitative measures and 0.908 for qualitative measures.

### Sample description and prevalence of SDC

**Fig 1:**
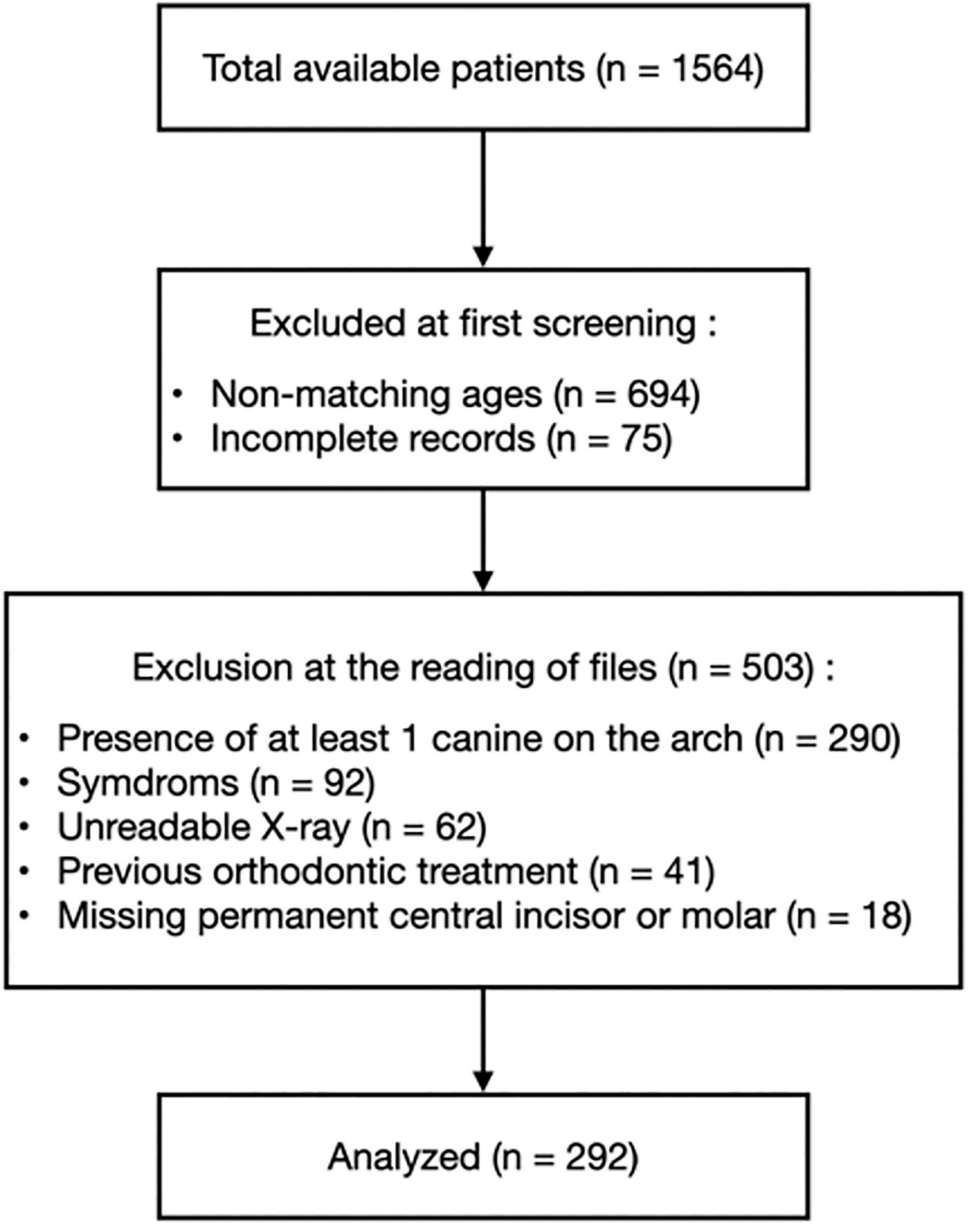
Flow chart for patient identification and analysis.

A total of 1564 records were reviewed leading to the inclusion of 292 patients i.e., 584 canines (Fig 1). The final sex ratio was 1:1, with girls being younger than boys, with respective ages of 9.0 ± 1.4 and 9.3 ± 1.4 (p < .05). Age ranges corresponding to the establishment of the permanent incisors (7–8 y.o.), the following stability phase (9–10 y.o.), and then the premolar setting phase (11–13 y.o.) were created (Table 1). SDC was detected in 39.0% of the patients included (114/292), and of 28.8% of the teeth considered (168/584) according to angulation [6], sector positioning (157) or both angulation and positioning [5]. Bilateral SDC was found in 54 children (18,5%), while 24 (8.2%) and 36 (12.3%) of them showed SDC on respectively the right or the left side only. SDC distribution was not associated to gender, age or occlusion relationship (Table 1).

**Table 1:** Description of study sample.

	Overall, N = 292^1^	SDC		P-value^2^
		No, N = 178^1^	Yes, N = 114^1^	
Sex ratio (F/M)	146/146	85/93	61/53	0.34
Mean age	9.1 ± 1.4	9.1 ± 1.4	9.2 ± 1.5	0.38
Age groups				0.31
[7–8]	106 (36.3%)	63 (35.4%)	43 (37.7%)	
[9–10]	137 (46.9%)	89 (50.0%)	48 (42.1%)	
[11–13]	49 (16.8%)	26 (14.6%)	23 (20.2%)	
Occlusal relationship				0.48
I	119 (40.8%)	69 (38.8%)	50 (43.9%)	
II.1	128 (43.8%)	78 (43.8%)	50 (43.9%)	
II.2	34 (11.6%)	22 (12.4%)	12 (10.5%)	
III	11 (3.8%)	9 (5.1%)	2 (1.8%)	

The composition of the sample analyzed was homogeneous. Neither gender, mean or dental age, nor occlusal relationships were related to SDC.

^1^ n (%); Mean ± standard deviation.

^2^ Pearson’s Chi-squared test; Two Sample t-test; Fisher’s exact test.

The mean angulation of the maxillary canines described by the α angle was 10.8 ± 8.5° and the mean distance from the cuspid point to the occlusal plane was 22.6 ± 11.5 mm (Table 2). For each, there was no significant difference between left and right side. More than a quarter of the canines (n = 162; 27.7%) were not located in sector 1. Primary canines were present in 507/584 cases (86,8%). Their presence on the arch was more frequently associated to SDC (p<0.05): 155/168 cases (92.3%) versus 352/416 (84.6%).

**Table 2:** Distribution of the 584 canines according to α angle, distance to occlusal plane and sector.

	Overall, N = 584^1^	α angle	
		≤ 31°, N = 573^1^	> 31°, N = 11^1^
Distance (mm)	22.6 ± 11.5	22.4 ± 11.3	30.8 ± 15.6
Angulation (degrees)	10.8 ± 8.5	10.1 ± 6.1	47.2 ± 23.9
Sector			
1	422 (72.3%)	416 (72.6%)	6 (54.5%)
2	132 (22.6%)	130 (22.7%)	2 (18.2%)
3	28 (4.8%)	26 (4.5%)	2 (18.2%)
4	2 (0.3%)	1 (0.2%)	1 (9.1%)

The data were such that few teeth showed strong angulation. Inclusion was suspected mainly because of radiographic superimposition on the lateral incisor root.

^1^ Mean ± standard deviation; n (%)

Three patients had lateral incisor agenesis. Of these, two were right-sided and one was bilateral. However, none of these patients showed SDC. Root resorption of a permanent lateral incisor resorption was detected in two patients on panoramic X-rays. The first case involved a left lateral incisor and was not associated with SDC. In the second case, both lateral incisors were resorbed with bilateral SDC. Only one maxillary first premolar showed resorption. The coronal mesio-distal diameters of the permanent central incisors and first permanent molars were significantly smaller in patients with SDC. Respectively 8.7 ± 0.6 mm versus 8.8 ± 0.7 mm (p < .05) for central incisors, and 10.0 ± 0.7 mm versus 10.2 ± 0.7 mm (p < .001) for molars. This difference was not significant for the lateral incisors: 5.6 ± 2.6 versus 6.0 ± 2.3 (p = .084). A reduced inter-molar width was also significantly associated to SDC: 38.7 ± 2.7 mm versus 39.4 ± 2.9 mm (p < .01). For parameters with a frequency of SDC greater than 10%, odds ratios were calculated. SDC was observed more frequently in case of reduced diameter central incisors and molars and also if inter-molar width was reduced (Table 3).

**Table 3:** Univariate regression analysis for SDC according to dental, occlusal parameters.

Dental, occlusal and age parameters	Overall	SDC « Yes »	OR^*1*^	95% CI^1^	P-value
Presence of primary canine	584	155	2.17	1.20, 4.22	**0.010**
Diameters (mm)					
Central incisor	584	168	0.70	0.53, 0.93	**0.013**
Lateral incisor	584	168	0.94	0.87, 1.01	0.089
Molar	584	168	0.62	0.46, 0.82	**<0.001**
Inter molar width	584	168	0.91	0.86, 0.97	**0.006**

The presence of primary canines, reduced mesio-distal diameters of central incisors and molars, and reduced palatal width were significantly associated with SDC on univariate analysis.

^1^ OR = Odds Ratio, CI = Confidence Interval

### Does SDC influence treatment planning?

Extractions of primary canines and premolars were significantly more frequent in cases of SDC (Table 4). Maxillary expansion by hyrax was indicated more frequently with SDC, without being significant. The strength of the association between therapeutic choice and SDC was established by calculating odds ratios (Table 4).

**Table 4:** Frequency and univariate regression analysis for SDC by orthodontic treatment plan options.

Orthodontic options	Overall	SDC		OR^1^	95% CI^2^	P -value
		No, N = 416^1^	Yes, N = 168^1^			
Primary canine extraction	584		61	3.32	2.19, 5.04	**<0.001**
No		355 (85.3%)	107 (63.7%)			
Yes		61 (14.7%)	61 (36.3%)			
Premolar extraction	584		40	1.66	1.06, 2.57	**0.027**
No		350 (84.1%)	128 (76.2%)			
Yes		66 (15.9%)	40 (23.8%)			
Hyrax expansion	584		151	1.53	0.88, 2.77	0.13
No		61 (14.7%)	17 (10.1%)			
Yes		355 (85.3%)	151 (89.9%)			

When SDC appeared to clinicians, they reported more extraction of primary canines and premolars.

^1^ n (%)

^2^ OR = Odds Ratio, CI = Confidence Interval

## Discussion

Interceptive treatment and therefore early diagnosis, help decrease the risk of impaction. They reduce the cost and duration of treatment, decrease the risk of complications or adverse outcomes, and facilitate orthodontic mechanics [16]. Radiographic evaluation before 10 years of age is therefore recommended [12, 17].

This study had two objectives. First, to determine the prevalence of impaction risk in orthodontic patients at University Hospital of XXX. Second, to identify predictive oral characteristics and evaluate associated treatment choices. Linear and angular measurements can be performed precisely on the panoramic X-ray [18]. This is the ideal screening for patients of this age. However, 2D X-rays have many limitations: magnification, loss of information, overlap and distortion [19]. 3D x-rays avoid these problems but their systematization is in contradiction with the as low as reasonably achievable principle [20]. Cone beam computed tomography, which is recommended in cases of canine impaction associated with a risk of lateral or premolar resorption, was therefore not retained as a screening examination [21].

From the angular and sector measurements, Ericson and Kurol concluded the impaction potential of the canine. The more mesial and horizontal the canine, the worse the prognosis even with extraction of the primary canine [11]. This method makes it possible to identify up to 78% of SDC, all of which are classified in sector 2, 3 or 4 [15]. An angulation of 31 degrees or more from the midline significantly decreases the chance of eruption [14]. A prevalence of SDC of 39% was found in this cohort, which is above the 28.3–32.5% prevalence in adolescents reported in the literature [22, 23]. However, some of our patients may have had a panoramic X-Ray due to clinical signs that alerted the practitioners. This selection bias may have led to some overestimation of the prevalence of SDC. As previously described [22, 24], no difference was noted in the prevalence and distribution according to gender.

Root resorption of lateral permanent incisors and premolars was detected in a very limited number of cases (2/292). This prevalence is below that described previously in 82 patients [25] and may have been due to the age distribution in our population. Most of them were aged 10 or below. This is in accordance with previous findings [12].

Although in the order of tenths of a millimeter, reduced mesio-distal dimensions of central incisors and molars were significantly associated with SDC. The literature is not homogeneous about this: some do not find any difference [26, 27], while for others the entire dentition could be smaller [28]. Without access to a frontal X-ray for each patient, inter-molar width was used as an indicator of maxillary transverse insufficiency with all the limitations that this entails, especially with regard to therapeutic implications. The association of reduced inter-molar width with SDC still remains controversial in literature [3, 27, 29–33]. In our study, such an association was found (Table 3).

These results are consistent with those of Cacciatore and Arboleda-Ariza [34, 35]. Regarding occlusion, most patients were Angle class I or II.1 without significant association to SDC. Again, the literature is fluctuating, with some associating SDC with class II.2 [26] and others class I [27, 36]. According to our data, the reduces dimensions of the central incisors and molars as well as the palatal width would be factors leading clinicians to suspect SDC.

If SDC is observed by the orthodontist, a treatment plan is implemented to prevent impaction. Several options are available: extraction of primary teeth, premolars and expansion. Extraction of teeth 53 and 63 was on average three times more frequent if SDC. This result must be put in perspective with the increased frequency of persistence of these teeth if SDC. This procedure is known to increase the rate of spontaneous placement of permanent canines to 65.2% versus 36% otherwise [37]. The ideal age would be between 10 and 11 years [38]. Many cases of SDC have been described in the context of a tooth-arch discrepancy [39]. In severe cases, extraction of four premolars becomes necessary. In the present study they were indicated one and a half times more frequently if SDC. However, the mean age of the cohort is lower than the age at which orthodontists indicate these extractions. Therefore, this rate could be revised upward over time. Transverse discrepancy of the maxilla is often associated with canine impaction [40]. The treatment of choice is the expansion with Hyrax screws. Indeed, beyond the SDC cases, this technique improves the functional context. It is therefore not surprising that no association was found.

## Conclusion

As a matter of fact, few oral signs could help practitioners suspect SDC. This retrospective study suggests, through the study of 292 clinical records, that only decreased mesiodistal dimensions and decreased inter-molar widths could be clinical signs of SDC. The following points can be drawn from this study:

- The prevalence of SDC was 39% in a French Dental Hospital 7-13-year-old population as evaluated using panoramic X-Ray.
- Clinical observation and panoramic X-ray remain a reference method to set up treatment planning to limit canine impaction.
- Reduced diameter of maxillary central incisors and first permanent molars as well as reduced inter-molar width were predictive factors.
- In cases of severe tooth-arch discrepancy, SDC led to the extraction of premolars.
- In cases of SDC, primary canines’ extractions were indicated at the age of 10 years.

## Supporting information

S1 Dataset (ZIP)
